# Pioglitazone Ameliorates Gentamicin Ototoxicity by Affecting the TLR and STAT Pathways in the Early Postnatal Organ of Corti

**DOI:** 10.3389/fncel.2020.566148

**Published:** 2020-10-29

**Authors:** Marijana Sekulic-Jablanovic, Matthew B. Wright, Vesna Petkovic, Daniel Bodmer

**Affiliations:** ^1^Department of Biomedicine, University of Basel, Basel, Switzerland; ^2^Strekin AG, Basel, Switzerland; ^3^Clinic for Otolaryngology, Head and Neck Surgery, University Hospital Basel, Basel, Switzerland

**Keywords:** inflammation, ototoxicity, gentamicin, cochlea, PPARγ

## Abstract

Noise trauma, infection, and ototoxic drugs are frequent external causes of hearing loss. With no pharmacological treatments currently available, understanding the mechanisms and pathways leading to auditory hair cell (HC) damage and repair is crucial for identifying potential pharmacological targets. Prior research has implicated increased reactive oxygen species (ROS) and inflammation as general mechanisms of hearing loss common to diverse causes. Novel targets of these two key mechanisms of auditory damage may provide new paths toward the prevention and treatment of hearing loss. Pioglitazone, an oral antidiabetic drug from the class of thiazolidinediones, acts as an agonist of the peroxisome proliferator-activated receptor-gamma (PPAR-γ) and is involved in the regulation of lipid and glucose metabolism. PPAR-γ is an important player in repressing the expression of inflammatory cytokines and signaling molecules. We evaluated the effects of pioglitazone in the mouse Organ of Corti (OC) explants to characterize its influence on signaling pathways involved in auditory HC damage. The OC explants was cultured with pioglitazone, gentamicin, or a combination of both agents. Pioglitazone treatment resulted in significant repression of interferon (IFN)-α and -gamma pathways and downstream cytokines, as assessed by RNA sequencing and quantitative PCR gene expression assays. More detailed investigation at the single gene and protein level showed that pioglitazone mediated its anti-inflammatory effects through alterations of the Toll-like receptor (TLR) and STAT pathways. Together, these results indicate that pioglitazone significantly represses IFN and TLR in the cochlea, dampening the activity of gentamicin-induced pathways. These data support our previous results demonstrating significant protection of auditory HCs in the OC explants exposed to pioglitazone and other PPAR-targeted agents.

## Introduction

Hearing loss is one of the most common disabilities and carries a large social and economic impact. Sensorineural hearing loss is caused by impairment and degradation of the sound-converting and -conducting structures of the cochlea, primarily cochlear hair cells (HCs) and spiral ganglion neurons (Nakagawa, 2014). Noise trauma, infection, and ototoxic drugs are common extrinsic causes of hearing loss. No satisfactory pharmacological treatments are available for hearing loss. To identify novel pharmacological targets, an improved understanding of the pathways leading to auditory HC damage and repair is crucial.

The inflammatory response in the cochlea develops as a result of pathogens or toxic insults mediated by drugs, noise, or immune challenges (Rock et al., 2010; Kalinec et al., 2017). Auditory HC damage occurs because of the overproduction of reactive oxygen species (ROS) and pro-inflammatory cytokines (Priuska and Schacht, 1995; Sergi et al., 2006). Several ototoxic drugs induce cell apoptosis and inflammation in the cochlea, directly or through the generation of ROS (Kaur et al., 2011; Oh et al., 2011). Trauma from acute or chronic noise exposure accelerates hearing loss through induction of the inflammatory response in the inner ear (Fujioka et al., 2006). The identification of therapies targeting both aspects of cochlear HC damage may offer new opportunities to reduce the impact of hearing loss.

Peroxisome proliferator-activated receptors (PPARs) belong to the nuclear receptor family of transcription factors and play key roles in biological signaling pathways that regulate lipid oxidation and synthesis, adipocyte differentiation, insulin action, cell proliferation, and inflammation. Based on these activities, small molecule drugs including fibrates and thiazolidinediones have been used in the treatment of diabetes and lipid abnormalities for more than 20 years (Orasanu et al., 2008). PPAR-γ exerts substantial anti-inflammatory effects through repression of multiple inflammatory cytokines (Martin, 2010; Croasdell et al., 2015). We previously reported that PPAR-γ is highly expressed in major cochlear structures, including inner and outer HCs, supporting cells, the stria vascularis, spiral ganglion, and cochlear nerve. We also demonstrated that the PPAR-γ-selective agonist pioglitazone and other small molecule PPAR agonists could preserve HCs from gentamicin-induced ototoxicity by preventing ROS induction and dampening inflammatory signaling (Sekulic-Jablanovic et al., 2017). These data are in agreement with earlier findings that intratympanic application of pioglitazone blocks oxidative stress and inflammatory signaling resulting from acute noise trauma in rats (Paciello et al., 2018).

In this report, we extend our investigation of pioglitazone signaling pathways in the organ of Corti (OC) explants, focusing on pathways of auditory HC protection. We cultured mouse OC explants in the presence of gentamicin to induce HC loss and compared RNA expression signatures with those of explants exposed to pioglitazone or a combination of both agents. We found that pioglitazone substantially inhibited the toxicity of gentamicin by broad downregulation of immune/inflammatory pathways, as determined by global RNA sequencing and quantitative (q)PCR. More detailed follow-up with single gene and protein analysis of key signaling molecules revealed that downregulation of multiple components of the Toll-like receptor (TLR) and STAT pathways mediated most of these effects. Our results indicate that in addition to the previously reported antioxidative mechanism, pioglitazone-mediated protection of cochlear HCs involves a substantial PPAR-γ anti-inflammatory component achieved through modulation of the TLR and STAT pathways, which are known to interact through cross-talk in TLR-induced inflammation (Luu et al., 2014).

## Materials and Methods

### Animal Care and Handling

Experiments were performed on OCs isolated from 5-day-old C57BL/6N mouse pups of both sexes obtained from Janvier Labs, France. All animals were maintained on a 12-h light/12-h dark schedule and had free access to water and a standard feeding regime. Animals were inspected regularly for health status. All animal procedures were conducted in compliance with the European Communities Council Directive of 24 November 1986 (86/609/EEC) and were approved by the Kantonales Veterinäramt, Basel, Switzerland.

### OC Tissue Culture and Drug Treatment

OC explants were isolated according to previously described methods (Sobkowicz et al., 1993). Briefly, 5-day-old (P5) C57BL/6N mice of both sexes were decapitated, and cochlear microdissections were performed under a light microscope to isolate the OC. OCs were incubated for 24 h in culture medium (Dulbecco’s Modified Eagle Medium, supplemented with 30 U/ml penicillin, 1% N1 supplement, 10% fetal calf serum, 25 mM HEPES) at 37°C and 5% CO_2_. For RNA sequencing experiments, six OCs per condition were placed in separate wells of 12-well plates, and each experimental treatment was performed in triplicate. For whole-genome RNA sequencing, we used the 6- and 24-h treatment time points. OCs were exposed to 50 μM gentamicin, 10 μM pioglitazone, or a combination of both. Control samples were incubated in parallel in a culture medium without drugs. For subsequent qPCR experiments, the treatment time point was 24 h with the same experimental design and compound concentrations. We chose the 50 μM concentration of gentamicin based on previous experiments conducted to define the concentration that reproducibly causes approximately 50% loss of HCs (Sekulic-Jablanovic et al., 2017).

### Quantification of HCs

OCs were fixed in 4% paraformaldehyde in phosphate-buffered saline (PBS) and permeabilized by washes in PBS-T (0.1% Triton X-100 in PBS). Samples were then incubated for 40 min with a 1:100 dilution of Alexa Fluor 488-labeled phalloidin (Molecular Probes, Eugene, OR, USA) in PBS-T at 4°C. The explants were mounted on a slide with Mowiol after several rinses with PBS. HCs were identified by the presence or absence of phalloidin-stained stereociliary bundles and circumferential F-actin rings on the cuticular plate of the outer HCs (OHCs) and inner HCs (IHCs). Only viable HCs were counted, with viability criteria requiring the presence of an intact cuticular plate with an intact stereociliary bundle. Cell populations were assessed using a fluorescence microscope (Olympus IX71) with images captured with an AxioCam system (Zeiss, San Diego, CA, USA). The right objective lens was marked with a 0.17-mm calibrated scale imposed on the field for reference. The single row of IHCs and all three rows of OHCs were longitudinally oriented within each 0.17-mm frame. Each successive 0.17-mm field was evaluated for the absence of IHCs and OHCs, beginning at the apex, and moving along the OC to the base. Segments containing 60 OHCs associated with 20 IHCs in a given microscopic field were included in the quantitative analyses. IHCs and OHCs were quantified, and these values were used to calculate percentage cell survival. The average number of OHCs and IHCs was determined for each individual explant by counting HCs in three segments selected randomly from the basal turn and three segments from the apical turn. Each treatment group included five mice, with three experimental replicates, for a total of 15 per treatment group.

### RNA Sequencing

#### RNA Integrity and Quantification

RNA was isolated from six OCs per condition, and each experimental treatment was performed in triplicate. The Direct-Zol RNA MiniPrep kit (Zymo Research, USA) was used for isolation according to the manufacturer’s instructions. For whole-genome RNA sequencing, 6- and 24-h treatment time points were used. RNA quality was assessed on a Bioanalyzer instrument (Agilent Technologies, Santa Clara, CA, USA) using the RNA 6000 Nano Chip (Agilent Technologies, Santa Clara, CA, USA, Cat# 5067-1511). Quantification was done by fluorometry using the QuantiFluor RNA System (Cat# E3310, Promega, Madison, WI, USA).

#### Library Preparation

Library preparation was performed with 200 ng total RNA using the TruSeq Stranded Total RNA Library Prep Gold (Cat# 20020598, Illumina, San Diego, CA, USA). Libraries were quality checked on a Fragment Analyzer (Advanced Analytical, Ames, IA, USA) using the Standard Sensitivity NGS Fragment Analysis Kit (Cat# DNF-473, Advanced Analytical). The results revealed excellent quality and homogeneity of libraries (average concentration was 121 ± 9 nmol/l, and the average library size was 356 ± 9 base pairs). Samples were pooled to equal molarity and quantified using the QuantiFluor ONE dsDNA System (Cat# E4871, Promega, Madison, WI, USA). Sample concentrations were adjusted to 1.4 pM for clustering on the NextSeq 500 instrument (Illumina). Samples were sequenced as single-read 76 bases (also: eight bases for index 1 and eight bases for index 2) using the NextSeq 500 High Output Kit 75-cycles (Illumina, Cat# FC-404-1005). Primary data analysis was performed with the Illumina RTA version 2.4.11 and Basecalling Version bcl2fastq-2.20.0.422. In total, an average of 46.9 ± 0.4 million reads per sample passed Illumina filtering.

#### RNA Sequencing Analysis

Reads were mapped to the mouse genome assembly^1^, with RNA-STAR (version 2.5.2a) default parameters. Gene expression data analysis was done using the R software package. RefSeq mRNA coordinates from the University of California, Santa Cruz^1^, and the qCount function from the QuasR package (version 1.16.0) were used to quantify gene expression as the number of reads that started within any annotated exon of a gene. The generalized linear model framework in the edgeR package (version 3.18.1) was used to identify differentially expressed genes.

### qPCR Protocol and RNA Isolation

OCs intended for RNA isolation were stored in RNAlater (Ambion, USA). RNA was isolated with a Direct-Zol RNA MiniPrep kit (Zymo Research, USA) according to the manufacturer’s instructions. RNA was isolated from 18 OCs per treatment condition. The quantity and quality of isolated RNA were determined with a NanoDrop 1000 (Thermo Fisher Scientific, Waltham, MA, USA), and the 260/280-nm absorbance ratios were between 1.8 and 2.1 for all samples. Total RNA (1,000 ng) was reverse transcribed into cDNA using a High-Capacity cDNA Reverse Transcription Kit (Applied Biosystems, USA). qPCR was done in triplicate and performed with an ABI Prism 7900HT Sequence Detection System (Applied Biosystems, USA) and with Power Sybr Green Master Mix (Applied Biosystems, USA). Primers used for qPCR were synthesized by Microsynth (St. Gallen, Switzerland). The primer sequences were (5′ to 3′): *Tlr4*, forward—ATG GCA TGG CTT ACA CCA CC and reverse—GAG GCC AAT TTT GTC TCC ACA; *Tlr2*, forward—CTC TTC AGC AAA CGC TGT TCT and reverse—GGC GTC TCC CTC TAT TGT ATT G; protein inhibitor of activated STAT1 (*Pias1*), forward—ACG CAA ACA CGA ACT TCT TAC A and reverse—TCC GCA GGC GTC ATA ATT TTC; *Stat1*, forward—TCA CAG TGG TTC GAG CTT CAG and reverse—GCA AAC GAG ACA TCA TAG GCA; *Myd88*, forward—TCA TGT TCT CCA TAC CCT TGG T and reverse—AAA CTG CGAGTGGGGTCAG; *Irf3*, forward-AACCGGAAAGAAGTGTTGCG and reverse—CCC TGG AGT CAC AAA CTC ATA C; *Irf7*, forward—TCC AGT TGA TCC GCA TAA GGT and reverse—CTT CCC TAT TTT CCG TGG CTG; and glyceraldehyde 3-phosphate dehydrogenase (*Gapdh*), forward—TGA CCT CAA CTA CAT GGT CTA CA and reverse -CTT CCC ATT CTC GGC CTT G. The final primer concentration was 250 nM per reaction. The thermocycling parameters were 10 min at 95°C, then 40 cycles of 95°C for 15 s and 60°C for 60 s. Template-free controls ensured that nonspecific amplification and DNA contamination could be excluded. The relative quantities of specifically amplified cDNAs were calculated with the comparative threshold cycle method, and *Gapdh* expression levels were used as the endogenous reference.

### Western Blotting

Five-day-old mouse pups of both sexes were sacrificed by decapitation, and their cochleae were carefully microdissected in ice-cold PBS. Isolated OCs were placed in cell lysis buffer (Sigma–Aldrich, Cat#C3228) with a protease inhibitor cocktail (Sigma–Aldrich, Cat#C3228, Cat#P8340), and then homogenized for 1 min on ice. Protein concentrations were determined by the Bradford method using Bio-Rad Protein Assay Dye Reagent Concentrate (Bio-Rad, Cat#5000006). Protein concentrations were confirmed using a NanoDrop (Thermo Fisher Scientific, Waltham, MA, USA). Samples were mixed with Laemmli sample buffer (Sigma–Aldrich, Cat#S3401), heated at 95°C for 5 min, and then resolved on 4%–20% Mini-PROTEAN^®^ TGX™ precast protein gels (Bio-Rad, Cat#4561096). For each sample, 10 cochlea explants (from five mice) were pooled and 10 μg protein loaded per lane. The experiment was replicated three times. After electrophoresis, separated proteins were transferred onto polyvinylidene fluoride membranes. Membranes were first incubated with 5% non-fat dry milk dissolved in PBS-T for 1 h at room temperature to block nonspecific protein binding sites. Next, the membranes were washed with PBS-T (3 × 10 min) and then incubated overnight at 4°C in 5% non-fat dry milk in PBS with one of the following primary antibodies: mouse polyclonal anti-Irf7 (Sigma–Aldrich, Cat#PRS3941), rabbit monoclonal anti-phospho-Irf7 (Cell Signaling, Cat#24129), rabbit monoclonal anti-PIAS1 (Cell Signaling, Cat#3550T), and rabbit polyclonal anti-β-actin (1:2,000, Cell Signaling Cat#4967). The membranes were then washed with PBS-T (3 × 10 min) and incubated for 1 h at room temperature with the appropriate horseradish peroxidase-conjugated secondary antibody (RRID:AB_2099233, RRID:AB_330924). After washing, immunoreactive protein bands were visualized using Super Signal West Dura Extended Duration Substrate (Thermo Fisher Scientific, Waltham, MA, USA, Cat#34076). An anti-β-actin antibody was used as a control to demonstrate equivalent protein loading. The intensity of the immuno-positive bands was determined using Fiji-win 32 software, capturing the identical regions on each blot, deducting the background signal. The intensity of all proteins of interest was normalized to the intensity of β-actin in the same sample.

### Immunofluorescence

The isolated OCs were fixed in 4% paraformaldehyde (Sigma–Aldrich, Cat# 158127) in PBS (Sigma–Aldrich, Cat# P4417), permeabilized by washing with 0.1% Triton X-100 (Sigma–Aldrich, Cat#X100) in PBS, and then incubated 1 h at room temperature with rabbit monoclonal anti-phospho-Irf7 (Cell Signaling, Cat#24129). Following a 1-h incubation, samples were washed and goat anti-rabbit Alexa Fluor 647 sary antibody (Thermo Fisher Scientific, Waltham, MA, USA, Cat#A21245) was added for 1 h at room temperature, plus 1:100 diluted solution of Alexa Fluor 488–labeled phalloidin (Molecular Probes, Cat#A12379) in PBS for 40 min at room temperature. Samples were washed with PBS and incubated with DAPI for 5 min. The OCs were then washed with PBS and mounted on microscope slides with Mowiol (Sigma–Aldrich, Cat#475904) for visualization and image capture.

### Statistical Analysis

Statistical calculations were performed using GraphPad Prism software (San Diego, CA, USA) using a two-way analysis of variance for comparison of multiple groups, or an unpaired *t*-test (non-parametric Mann–Whitney test) for comparison of two groups in case of HC count. Data were confirmed to be normally distributed by conducting a Shapiro-Wilk test. No outliers were detected at the significance level of 0.05 (two-sided). GraphPad Outlier calculator which performs Grubbs test, also called the ESD method, was used to perform this test. *P*-values are presented in the figure legends.

## Results

### Pioglitazone Blocks Gentamicin-Induced Toxicity to Auditory HCs

**Figure 1:**
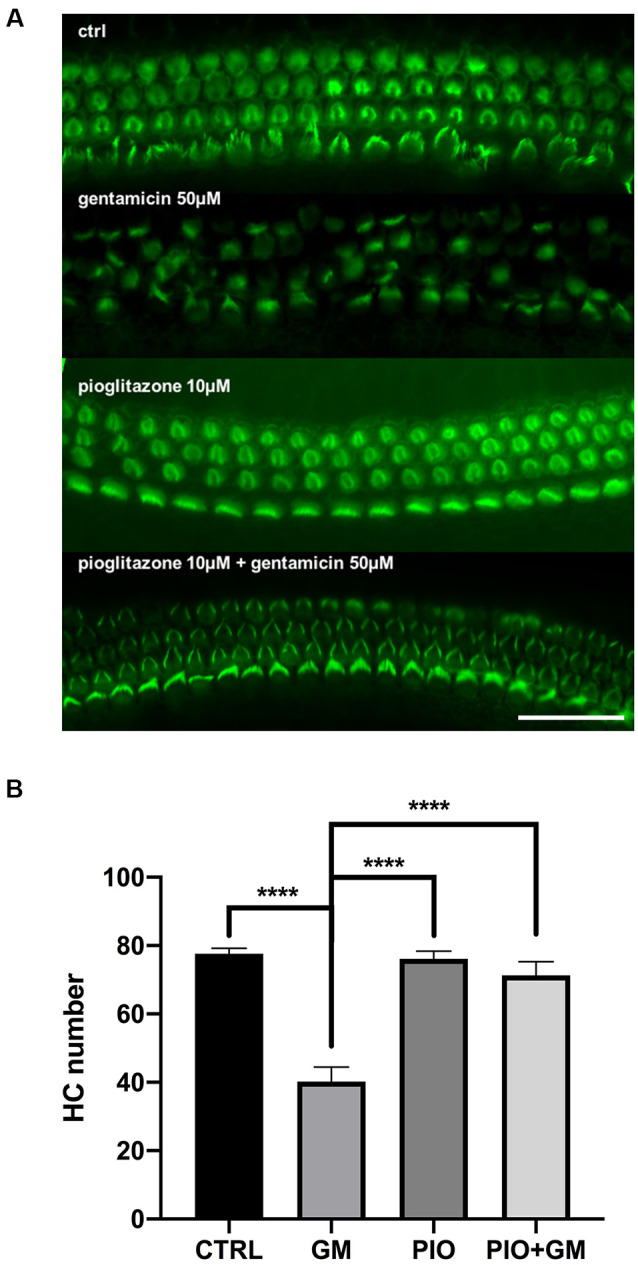
Pioglitazone preserves the cochlear hair cell (HC) number in the gentamicin-exposed organs of Corti (OC) explants. **(A)** Fluorescent micrographs showing green phalloidin-stained mouse OC explants, exhibiting three orderly rows of intact outer HCs and a single row of inner HCs in the basal cochlear turns of control cultures. Treatment with Ten micromolar pioglitazone (PIO) resulted in almost complete HC protection following gentamicin (GM) treatment. Ten micromolar pioglitazone alone did not cause any loss of HCs. OCs cultured with 50 μM gentamicin for 24 h showed a consistent 50 ± 10% loss of HCs. **(B)** Quantitative data on HC survival. Data are expressed as the mean (±SEM) numbers of surviving outer OHCs and inner HCs (IHCs) in standard segments. Data were obtained from 10 explants (five mice) per group, from both sexes, in triplicate; *n* = 15 mice per treatment group. Stars represent statistical difference of GM compared to all other treatments; ****p < 0.0001. Scale bar, 50 μm.

We previously reported that multiple selective and dual-active agonists (muraglitazar, tesaglitazar, Fenofibric acid, and pioglitazone) of the PPAR-γ and PPAR-α nuclear hormone receptors dramatically blocked aminoglycoside-induced HC apoptosis and loss in mouse OC explants in culture (Sekulic-Jablanovic et al., 2017). All drugs were active, with the extent and dose-dependence for the prevention of gentamicin toxicity dependent on the specific molecule. In the current study, we further explored the signaling pathways by which the PPAR-γ agonist pioglitazone, which showed the most robust activity, mediates its effects. Mouse OCs treated with 50 μM gentamicin alone exhibited severe HC toxicity, reflected by loss of approximately 50% of HCs, as detected by the absence of phalloidin-stained stereociliary bundles when compared to control samples (Figure 1A). Pioglitazone at 10 μM completely prevented the toxic effects of gentamicin, reflected in almost complete preservation of HCs in OCs cultured with both agents (Figure 1B). We employed these concentrations and experimental protocol for the following exploration of gene expression signatures and signaling pathways.

### mRNASeq Analysis Reveals That Pioglitazone Blocks Gentamicin Induction of Inflammatory Gene Signatures in Cultured OCs

The OC lacks immune cells but has the capacity for stress-induced upregulation of immune signaling in response to various insults including ototoxic agents or noise trauma (Cai et al., 2014). RNA sequencing identified a total of 14,701 genes expressed in the mouse OC. A comparison of gene signatures between gentamicin with and without pioglitazone revealed significant modulation of multiple pathways in cellular immune response and inflammation (Table 1). We found that both the interferon (IFN)α and IFNγ pathways were strongly induced by gentamicin at both early (6 h) and late (24 h) time points. In contrast, pioglitazone treatment of OCs compared to controls had no effect on the IFNα response at 6 h but led to a significant downregulation of IFNα at 24 h. IFNγ response gene signatures were downregulated by pioglitazone at both the 6 and 24-h time points when compared to control. Combined treatment of pioglitazone and gentamicin compared to gentamicin treatment alone showed no changes at 6 h for IFNα and IFNγ response but a remarkable decrease after 24 h. The inflammatory response stayed consistently decreased in the presence of pioglitazone, whether it was compared to control or to gentamicin-only treatment. Of interest, significant changes occurred at the level of the IL2/6JAK-STAT pathways, with the presence of pioglitazone consistently leading to decreased gene expression compared to control or gentamicin treatment. The most prominent effect of gentamicin, stimulation of the inflammatory pathway, was counteracted in the presence of pioglitazone.

**Table 1:** Gentamicin (GM) upregulates inflammatory pathways in hair cells (HCs), whereas pioglitazone (PIO) counteracts this effect.

Pathways	Genes	GM vs. CTRL		PIO vs. CTRL		PIO + GM vs. GM	
		**P**					
		6 h	24 h	6 h	24 h	6 h	24 h
Interferon alpha response	84	3.291e-47	8.553e-11	(0.49000)	1.317e-05	(0.277085)	3.664e-09
Interferon gamma response	175	1.086e-19	5.662e-05	5.198e-07	1.712e-05	(0.5718)	7.695e-07
Inflammatory response	140	(0.4574)	2.491e-01	1.272e-05	8.592e-06	3.682e-06	1.201e-01
IL6 JAK STAT3 signaling	68	(0.3165)	3.344e-02	1.515e-02	(0.06813)	9.656e-02	7.307e-03
IL2 STAT5 signaling	171	2.042e-01	(0.3168)	4.007e-04	1.877e-03	1.410e-01	6.884e-04

*Pathway analysis of RNA sequencing results of 6 and 24-h treatments with a comparison between selected treatment conditions. Numbers represent *p*-values. Blue represents upregulation, red indicates downregulation and parentheses mark results that were not statistically significant*.

### Downregulation of IFN Target Genes After Pioglitazone Treatment

**Figure 2:**
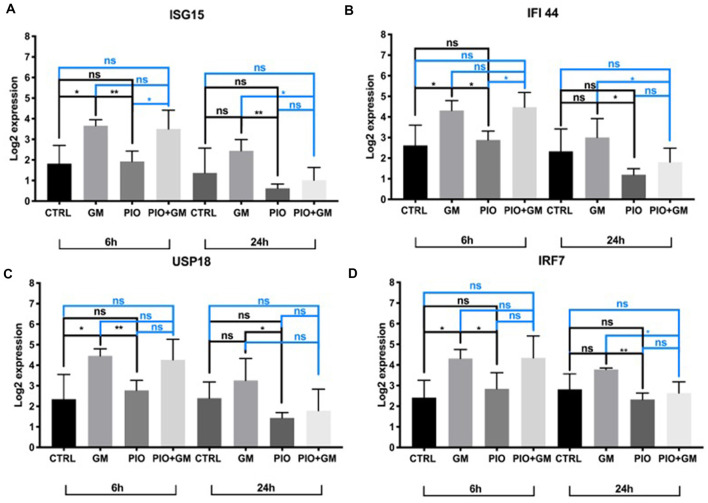
Isg15, Ifi44, Usp18, and Irf7 expression is downregulated in the presence of pioglitazone. **(A)** Gentamicin (GM) increased *Isg15* expression after 6 h when comparing to control or pioglitazone (PIO), combined treatment did not show any changes at this time point when compared to control or gentamicin, and was increased compared to pioglitazone; pioglitazone’s effect was significant at 24 h in downregulating Isg15 expression alone or in combination with gentamicin when compared to gentamicin alone. **(B)** Gentamicin upregulated *Ifi44* expression at 6 h, whereas pioglitazone alone or in combination with gentamicin kept *Ifi44* expression levels significantly lower when compared to gentamicin alone at 24 h. **(C)** Usp18 expression was upregulated by gentamicin at 6 h compared to control or pioglitazone, and at 24 h of treatment, there was a significant difference between gentamicin and pioglitazone in which the latter decreased *Usp18* levels. **(D)** *Irf7* expression was upregulated after 6 h of exposure and significantly differed when compared to control or pioglitazone. At 24 h, pioglitazone decreased *Irf7* expression levels alone or in combination with gentamicin when compared to gentamicin alone. Six OCs per condition were used, and each experimental treatment was performed in triplicate *n* = 3; *p < 0.05, **p < 0.01, ns (not significant).

IFNs induce activation of expression of numerous transcripts involved in immunity and inflammation response. In this study, we identified several of these genes as being affected by gentamicin and pioglitazone. IFN-stimulated gene 15 (*Isg15*) was directly upregulated in the presence of gentamicin, and pioglitazone together with gentamicin suppressed this effect at 24 h of treatment (Figure 2A). Pioglitazone alone showed a significant difference when compared to gentamicin-only treatment at 6 and 24 h and did not increase *Isg15* expression when compared to control levels for both time points*. Ifi44* and *Irf7* showed a similar pattern of expression at 6 h, with gentamicin inducing significant upregulation of their expression and pioglitazone resulting in expression levels similar to those of controls (Figures 2B,D). Combined treatment with gentamicin and pioglitazone had no significant impact on the expression of these two genes at 6 h, but at 24 h pioglitazone alone or in combination with gentamicin significantly decreased expression of *Ifi44* and *Irf7* when compared to gentamicin alone. *Usp18* expression was upregulated by gentamicin at both time points, while pioglitazone treatment resulted in unchanged *Usp18* levels compared to control samples. Pioglitazone single treatment resulted in a significantly lower expression when compared to gentamicin at 6 and 24 h, but this effect did not persist under co-treatment with pioglitazone and gentamicin (Figure 2C). Also, we found that the number of pro-inflammatory genes and stress-induced genes decreased in HCs after pioglitazone treatment in the presence of gentamicin compared to gentamicin alone. These genes mostly encoded chemokines (*Ccl2, Ccl9, Ccl12, Cxcl10, Cxcl16*, and *Gbp2* with *Il-16*; Table 2). Overall, pioglitazone shows anti-inflammatory effects on the gene expression level that seem to be differently pronounced concerning the incubation time in cell culture.

**Table 2:** Pro-inflammatory and stress-induced genes are decreased in hair cells (HCs) after pioglitazone (PIO) exposure.

Gene	Gene name	PIO + GM vs. GM	
symbol		*p*	
Ccl2	Chemokine (C-C motif) ligand 2	0.0436678590	downregulated
Ccl9	Chemokine (C-C motif) ligand 9	0.008335264
Ccl12	Chemokine (C-C motif) ligand 12	0.020275026
Cxcl10	Chemokine (C-X-C motif) ligand 10	0.02378565
Cxcl16	Chemokine (C-X-C motif) ligand 16	0.003689454
Gbp2	Guanylate binding protein 2	0.01283124
Il-16	Interleukin 16	2.398601e-05	

*Table with the most significant gene expression changes of RNA sequencing results and their p-values in samples exposed to a combination of pioglitazone and gentamicin (GM) compared to gentamicin alone (*n* = 9)*.

### Pioglitazone Affects the TLR and STAT Pathways in the Cochlea

**Figure 3:**
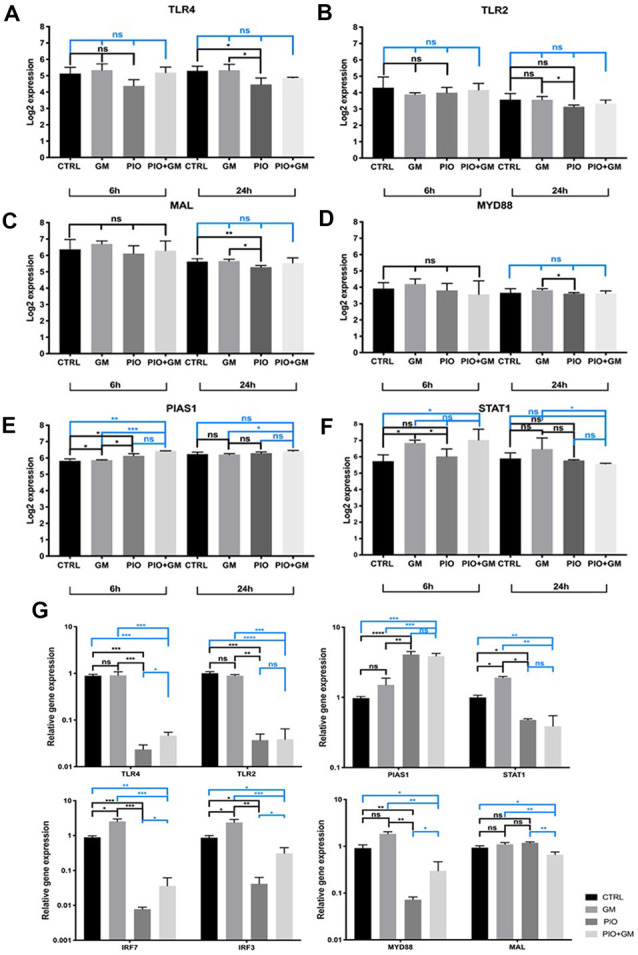
Pioglitazone affects the gene expression of Toll-like receptor (TLR) and STAT pathway components. **(A–F)** RNA sequencing results for 6 h and 24 h treatment at the single-gene level. **(A,B)** A decrease in the *Tlr4* and *Tlr2* receptor expression in samples exposed to pioglitazone (PIO) was evident after 24 h of treatment. **(C,D)** *Mal* and *Myd88*, initial components of the TLR pathway, were affected by pioglitazone treatment at 24 h and significantly downregulated when compared to gentamicin treatment or control (CTRL). **(E)** A significant increase in *Pias1* expression under the influence of pioglitazone in combination with gentamicin (GM), when compared to gentamicin alone, was evident at both time points. **(F)** *Stat1* expression was decreased by single pioglitazone treatment at 6 h, but combined treatment with pioglitazone and gentamicin triggered a significant decrease only after 24 h when compared to gentamicin alone. Six OCs per condition were used, and each experimental treatment was performed in triplicate *n* = 3; *p < 0.05, **p < 0.01, ***p < 0.001, ns (not significant). **(G)** qPCR validation of gene expression changes observed after RNA sequencing. The results shown are for 24-h treatment. Obvious results were obtained for *Tlr4*, *Tlr2*, *Myd88*, *Irf7*, and *Irf3*, with a significant decrease in samples treated with pioglitazone alone or in combination with gentamicin, compared to gentamicin only. Exceptions were no significant difference in *Stat1* expression in the presence of pioglitazone alone, and a decreased Mal expression only with the combination of pioglitazone and gentamicin. Results are the mean fold-change in transcript levels ± SD. *n* = 3; *p < 0.05, **p < 0.01, ***p < 0.001, ****p < 0.0001, ns (not significant).

A closer look at the RNA sequencing results at the single-gene level revealed a decrease in *Tlr4* and *Tlr2* receptor expression in samples exposed to pioglitazone after 24-h treatment (Figures 3A,B). We observed a similar pattern for *Mal* and *MyD88*, which are initial components of the TLR pathway (Figures 3C,D). Consistent with these results is a previously identified decrease in the expression of a downstream *Irf7* (Figure 2D), whose activation is regulated by the MyD88-dependent branch of the TLR pathway. Moreover, our qPCR results showed a decreased expression of *Irf3* in the presence of pioglitazone alone or combined with gentamicin, when compared to gentamicin alone or control (Figure 3G). We unexpectedly found no other changes in this branch of the TLR pathway, except for the downstream *Irf3* factor.

At the same time, *Pias1* expression increased under the influence of pioglitazone alone after 6 h treatment and in gentamicin combined treatment compared to gentamicin alone at 6 and 24 h (Figure 3E). PIAS1 has been described as negatively regulating the TRIF, IRF3, and IRF7 components of the TLR pathway and mainly STAT1 dimers. *Stat1* expression was decreased by pioglitazone treatment at 6 h, but combined treatment with pioglitazone and gentamicin yielded a significant decrease only after 24 h when compared to gentamicin-only treatment (Figure 3F). qPCR on samples treated for 24 h was performed to further examine the expression of these genes, and we obtained more obvious results in the cases of *Tlr4*, *Tlr2*, *Myd88*, *Irf7*, and *Irf3*, with a significant decrease under treatment with pioglitazone alone or in combination with gentamicin, compared to gentamicin only or control. *Stat1* and *Mal* expression levels showed to be decreased by pioglitazone alone or in combination with gentamicin when compared to gentamicin treated or control samples (Figure 3G). These results indicate that gentamicin as well as pioglitazone have distinctive effects on TLR and STAT pathways.

### PIAS1 Protein and Gene Expression Increases in the Presence of Pioglitazone

**Figure 4:**
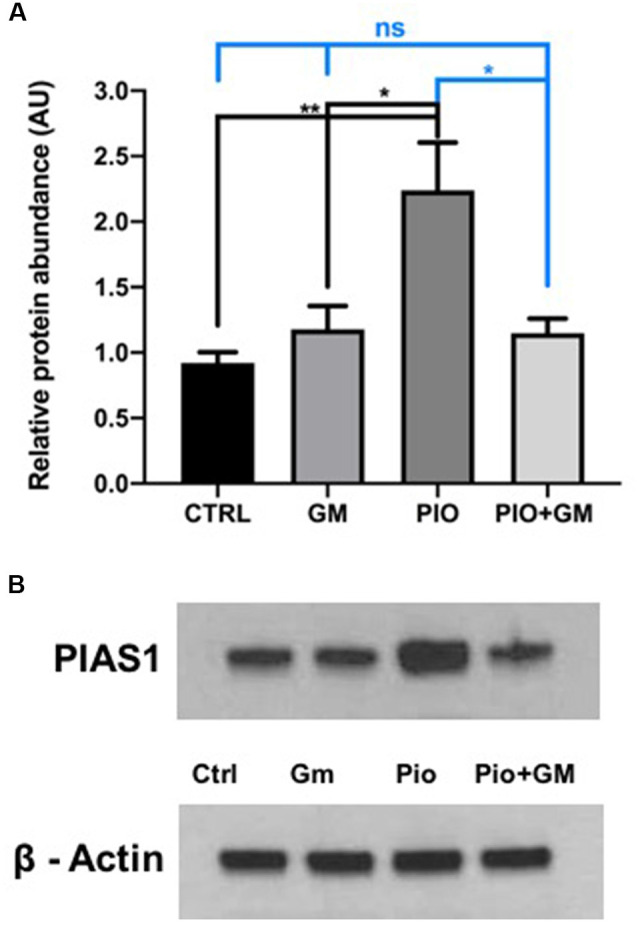
PIAS1 protein and gene expression increased in the presence of pioglitazone (PIO). **(A)** Western blot analysis of OC protein isolates revealed a significant increase in PIAS1 levels in samples treated with 10 μM pioglitazone compared to control (CTRL), gentamicin (GM)-exposed and PIO plus GM-treated samples. In combined treatment of 10 μM pioglitazone and 50 μM gentamicin, PIAS1 protein levels remained unchanged compared to control or gentamicin. **(B)** Representative western blot image showing increased PIAS1 protein presence in samples exposed to pioglitazone and no changes in protein levels in control, gentamicin, or combined treatment of pioglitazone and gentamicin. Data were obtained from 10 explants (five mice) pooled per treatment group, and done in triplicate, *n* = 3; *p < 0.05, **p < 0.01, ns (not significant). ß-actin was used as a loading control.

Western blot analysis of OC protein isolates revealed significant increases in PIAS1 levels in samples treated with 10 μM pioglitazone compared to control or gentamicin-exposed samples (Figure 4A). In combined treatment with 10 μM pioglitazone and 50 μM gentamicin, PIAS1 protein levels remained unchanged compared to control or gentamicin, as shown in Figure 4A and the representative western blot image (Figure 4B).

### Gentamicin Induces IRF7 Phosphorylation and Nuclear Translocation, and Pioglitazone Reverses This Effect

**Figure 5:**
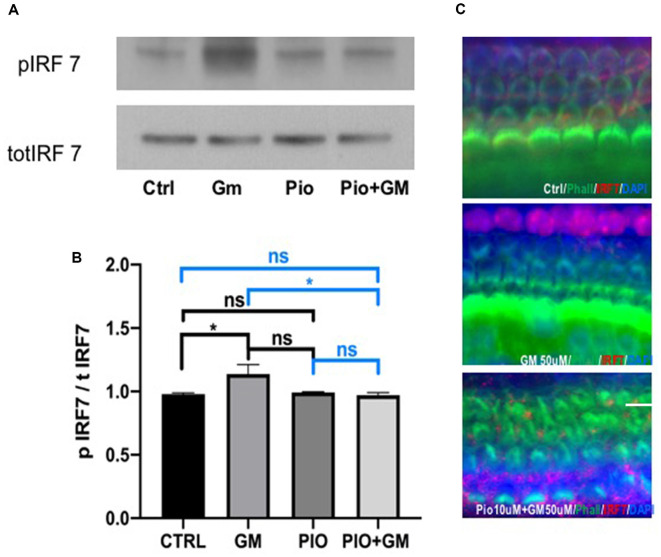
Pioglitazone (PIO) affects gentamicin (GM)-induced IRF7 phosphorylation and nuclear translocation. **(A)** Representative western blot of IRF7 phosphorylation. IRF7 phosphorylation was significantly increased in protein lysates of samples exposed to 50 μM gentamicin compared to control. Pioglitazone alone did not induce significant change compared to either control or gentamicin. Combined treatment of pioglitazone and gentamicin lowered the phosphorylation levels of IRF7 when compared to gentamicin. **(B)** Quantitative analysis of western blots. Values indicate the ratio of phospho-IRF7 and total-IRF7 expression levels. Data were obtained from 10 explants (five mice) pooled per treatment group and done in triplicate, *n* = 3. *p < 0.05, ns (not significant). **(C)** Because IRF7 phosphorylation induces its translocation to the nucleus, we immunostained cultured OCs to detect IRF7. IRF7 (red) was more concentrated in the nucleus together with DAPI in samples exposed to gentamicin, while in the control and pioglitazone-exposed OCs, the IRF7 remained mostly in the cytoplasm. Green represents phalloidin-stained HCs. Scale bar, 50 μm.

IRF7 phosphorylation was significantly increased in protein lysates of samples exposed to 50 μM gentamicin compared to control after 24 h incubation (Figures 5A,B). Pioglitazone alone did not result in a significant change compared to either control or gentamicin. Combined treatment with pioglitazone and gentamicin reduced levels of phosphorylated IRF7 when compared to gentamicin. Because IRF7 phosphorylation induces its translocation to the nucleus, we immunostained cultured OCs for IRF7 detection. Based on DAPI staining, IRF7 was more concentrated in the nucleus in samples exposed to gentamicin, whereas in the control and pioglitazone-exposed OCs, the IRF7 remained mostly in the cytoplasm (Figure 5C).

## Discussion

Inflammation in the ear resulting from exposure to ototoxic drugs or excessive noise is a unique case of immune reaction that is not pathogen provoked (Rock et al., 2010). In the cochlea, the immune response can be initiated by stress and cellular damage products through interaction with TLRs, RAGE (receptor for advanced glycation end-products), and other receptors (Peri and Calabrese, 2014; Keithley, 2018). The OC sensory cells are the main structures suffering damage after noise or ototoxic drug exposure and for a long time were considered to lack any immune function. Cai et al. (2014) showed that the cochlear sensory epithelium constitutively expresses immune/inflammatory-response related-genes. This result was also confirmed in our current study where, after exposure to ototoxic gentamicin or PPAR-γ agonist pioglitazone, major changes occurred in the IFN-alpha and -gamma and inflammatory pathways.

IFNs are multifunctional proteins affecting cell proliferation, cell differentiation, the apoptotic immune response, and inflammation. Here we explored the overall gene expression in OC and found the IFN-alpha and -gamma pathways to be significantly increased after gentamicin treatment in cultured explants. Aminoglycosides induce HC death, so an observed increase in the cytokine/inflammatory pathways could be the result of cellular damage products. Damage in supporting cells precedes damage in adjacent HCs and could be mediated by the inflammatory response. Indeed, supporting cells are the major site for the expression of immune genes (Cai et al., 2014).

PPAR-γ receptors suppress inflammatory cytokine production in other tissues (Youssef and Badr, 2004; Kapadia et al., 2008). In cochlea and OC, however, this effect was not previously described. Using OCs cultured with the PPAR-γ agonist pioglitazone, we show here for the first time the downregulation of cytokine-inducing pathways under this condition. Some of the genes linked to immune and inflammatory responses, which include cytokine–cytokine receptor interactions, the chemokine signaling pathway, the TLR signaling pathway, and the NOD-like receptor signaling pathway, have previously been connected with the cochlear response to acoustic overstimulation (Yang et al., 2016). In our study, the presence of pioglitazone, compared to gentamicin alone, led to decreased expression of several pro-inflammatory genes in OC, mostly chemokines. In clinical practice, steroid-based treatments against noise-induced hearing loss have been effective most likely because of an anti-inflammatory reaction that suppresses excessive inflammation. However, in some studies, large doses of steroids harmed long-term cochlear function (Karlidag et al., 2002; Takemura et al., 2004). This variable outcome of steroid treatment might be explained by the actions of multifunctional pro-inflammatory cytokines (Fujioka et al., 2006).

Of interest, *Cxcl10*, *Gbp2*, and *Isg15* all were downregulated in the presence of pioglitazone. All three are regulated by cRel, a transcription factor from the NFκB family. *Gbp2* is upregulated under conditions of cellular stress and can be expressed in response to interferons (Wood and Zuo, 2017). *Isg15* is a homolog of ubiquitin in vertebrates and is strongly upregulated following induction by type I IFN. Also, a multifunctional component of the IFN response*, Usp18*, whose expression is regulated by interferon-sensitive response element (ISRE) binding and activation, was upregulated in the samples exposed to gentamicin, whereas only pioglitazone alone treatment resulted in decreased *Usp18* expression when compared to gentamicin. Another component of the type I IFN-inducible gene family, called *Ifi44*, was increased in samples exposed to gentamicin, and in the presence of pioglitazone this effect was inverted. *Ifi44* is associated with inflammation in gentamicin-induced ototoxicity and nephrotoxicity (Hu et al., 2017).

Another interesting result was with *Tlr4* and *Tlr2* receptor expression, which was decreased in the presence of pioglitazone alone. The major effect was observed, however, in the expression of downstream components such as *Irf3* and *Irf7*, whose activation subsequently increases under IFN and IFN-inducible gene expression. This effect in turn would positively feed the IFN-IFNAR/IFNGR loop through the STAT1/STAT2–mediated pathway. Studies show that *Tlr4* knockout mice are not impaired in their hearing performance and that lack of this receptor becomes significant only after exposure to noise, where the absence of this receptor seems to be advantageous and otoprotective (Cai et al., 2014; Vethanayagam et al., 2016).

Several studies have emphasized TLR4 activation as one of the pathways leading to inflammation after noise or ototoxicity caused by aminoglycoside or cisplatin treatments. The specific ligand that activates TLR4 in these cases is so far unidentified, but the expression of *Tlr4* is increased in the cochlea as a response to these damaging factors (Oh et al., 2011). Results obtained in patients with sudden sensorineural hearing loss revealed that those with the most severe hearing loss had the highest *Tlr2* expression (Yang et al., 2015). In our study, a moderate decrease in *Tlr4*, *Tlr2*, *Mal*, and *Myd88* components in the presence of pioglitazone alone would not be likely to lead to complete protection from gentamicin toxicity and inflammatory damage; as combined treatment with pioglitazone and gentamicin did not show a significant difference in the expression of these genes in the RNAseq results, and a slight change after the qPCR testing. No other downstream components of this pathway were affected except *Irf3* and *Irf7*, whose translocation to the nucleus stimulates cytokine expression.

We found that the anti-inflammatory molecule PIAS1 was significantly upregulated in the presence of pioglitazone. Aside from directly inhibiting STAT1-mediated gene expression, PIAS1 has several different points of interference with pro-inflammatory pathways (Liu et al., 1998). PIAS1 interacts with IRF3 and inhibits the DNA binding activity of IRF3 (Li et al., 2013). A ligase within the PIAS family is responsible for the sumoylation of IRF3 and IRF7 observed after a viral infection, leading to the negative regulation of type I IFN gene expression, and PIAS1 is a mediator of IRF7 sumoylation (Kubota et al., 2008; Chang et al., 2009). Because *Irf7* expression depends on the activated IFN I and TLR pathways and IRF7 protein itself promotes IFN-alpha and *Irf7* gene expression by binding to ISRE, interference with its activation and nuclear translocation could lead to decreased *Irf7* mRNA levels. We have discovered increased phosphorylation of IRF7 protein in samples exposed to gentamicin, whereas pioglitazone-treated samples had levels similar to control samples. PIAS1 proved to be important in maintaining proper amounts of type I IFNs. Another group has shown that PIAS1 can inhibit TRIF-induced promoter activity and prevent the whole downstream activation of the TLR4 pathway (Kubota et al., 2011).

**Figure 6:**
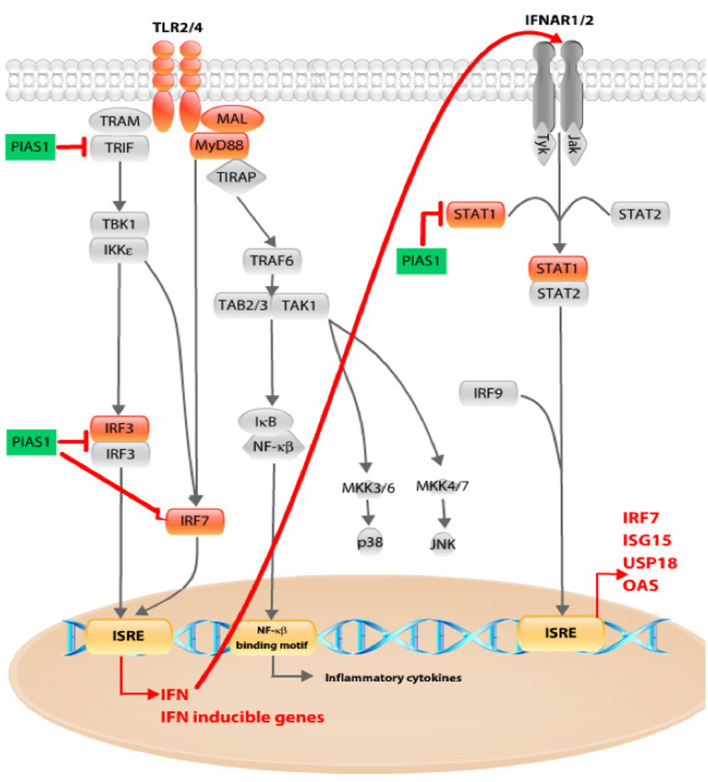
Schematic overview of pioglitazone effects on TLR and interferon (IFN) pathway. Pioglitazone decreased *Tlr4* and *Tlr2* receptor expression together with *Mal* and *Myd88* (marked in orange). The major effect was observed in decreased expression of downstream pathway components such as *Irf3* and *Irf7*, whose activation subsequently upregulates IFN and IFN-inducible gene expression, as found in our gentamicin-treated OCs. Pioglitazone upregulated *Pias1* (marked in green). PIAS1 can interact with IRF3 and inhibit its DNA-binding activity. PIAS1 acts as a mediator of IRF7 sumoylation. *Irf7* expression is dependent on the activated IFN I and TLR pathways. IRF7 protein itself promotes IFN-α and *Irf7* gene expression by binding to interferon-sensitive response element (ISRE), and interference with its activation and nuclear translocation could lead to decreased *Irf7* mRNA levels. We have discovered increased phosphorylation of IRF7 protein in samples exposed to gentamicin, whereas pioglitazone-treated samples had levels similar to control samples. The third point of PIAS1 interaction with the IFN pathway could be the blocking of STAT1 and subsequent activation of ISRE-mediated gene transcription. By interfering at any of these points, PIAS1 could decrease IFN and IFN-inducible gene expression, leading to inhibition of IFN-dependent IFN-pathway activation (red arrow).

We have proposed a scheme to summarize these PIAS1 effects and points of interference with the TLR and IFN pathways, with our results incorporated (Figure 6). Gentamicin boosts expression of genes related to the inflammatory response in OC explants, but pioglitazone counteracts this effect. Because the increased inflammatory response and ROS after noise exposure or ototoxic insult arise quickly in the cochlea and are directly associated with HC death, a drug that could prevent the earliest stages of inflammation and oxidative stress would be crucial for hearing preservation.

Our previous studies demonstrated the robust effects of pioglitazone to prevent auditory HC loss *in vitro* (Sekulic-Jablanovic et al., 2017). We have also shown that intratympanic administration of pioglitazone protects from acute noise-induced hearing loss *in vivo* (Paciello et al., 2018). Pioglitazone blocks the immediate increase in ROS and the more delayed induction of cochlear inflammatory responses. These data suggest that early intervention with pioglitazone before the administration of ototoxic drugs may be most effective.

Pioglitazone has previously been shown to penetrate the central nervous system. Orally administered pioglitazone was shown to enter the brains of transgenic mice representing a model of Alzheimer’s disease (AD) at sufficient concentrations to exert biologically relevant effects to reduce AD-related pathological burden and to suppress glial activation (Maeshiba et al., 1997; Heneka et al., 2005; Roberts et al., 2009). The combination of blood-brain barrier penetration and demonstrated activity on AD-related pathophysiology suggests that oral pioglitazone may also be useful in countering cochlear pathologies associated with ototoxic agents.

## Data Availability Statement

The original contributions presented in the study are publicly available. This data can be found here: https://www.ncbi.nlm.nih.gov/geo/query/acc.cgi?acc=GSE158431.

## Ethics Statement

The animal study was reviewed and approved by Kantonales Veterinäramt, Basel, Switzerland under licence number 2263.

## Author Contributions

MS-J conceived, designed and carried out the experiments, analysis, and wrote the manuscript with input from all authors. VP contributed to the interpretation of the results and image analysis. MW contributed to the interpretation of the results and writing the manuscript. DB critically revised the manuscript and did the final approval of the version to be published. All authors provided critical feedback and helped shape the research, analysis and manuscript. All authors contributed to the article and approved the submitted version.

## Conflict of Interest

MW was employed by the company Strekin AG, Basel (Switzerland). Prof. DB is a co-founder and holds shares in Strekin AG.

The remaining authors declare that the research was conducted in the absence of any commercial or financial relationships that could be construed as a potential conflict of interest.

## Footnotes

^1^http://genome.ucsc.edu/

## Abbreviations

AD: Alzheimer’s disease
HC: Hair cell
IFN: Interferon
IFNAR/IFNGR: Interferon-alpha receptor/Interferon-gamma receptor
IHC: Inner hair cell
OC: Organ of Corti
OHC: Outer hair cell
PIAS1: Protein inhibitor of activated STAT1
PPAR: Peroxisome proliferator-activated receptor
TLR: Toll-like receptors.

## References

[B1] Cai Q. Vethanayagam R. R. Yang S. Bard J. Jamison J. Cartwright D.. (2014). Molecular profile of cochlear immunity in the resident cells of the organ of corti. J. Neuroinflammation 11:173. 10.1186/s12974-014-0173-8 25311735 PMC4198756

[B2] Chang T.-H. Kubota T. Matsuoka M. Jones S. Bradfute S. B. Bray M.. (2009). Ebola zaire virus blocks type I interferon production by exploiting the host SUMO modification machinery. PLoS Pathog. 5:e1000493. 10.1371/journal.ppat.1000493 19557165 PMC2696038

[B3] Croasdell A. Duffney P. F. Kim N. Lacy S. H. Sime P. J. Phipps R. P. (2015). PPARγ and the innate immune system mediate the resolution of inflammation. PPAR Res. 2015:549691. 10.1155/2015/549691 26713087 PMC4680113

[B4] Fujioka M. Kanzaki S. Okano H. J. Masuda M. Ogawa K. Okano H. (2006). Proinflammatory cytokines expression in noise-induced damaged cochlea. J. Neurosci. Res. 83, 575–583. 10.1002/jnr.20764 16429448

[B5] Heneka M. T. Sastre M. Dumitrescu-Ozimek L. Hanke A. Dewachter I. Kuiperi C.. (2005). Acute treatment with the PPARgamma agonist pioglitazone and ibuprofen reduces glial inflammation and abeta1-42 levels in APPV717I transgenic mice. Brain 128, 1442–1453. 10.1093/brain/awh452 15817521

[B6] Hu J.-G. Fu Y. Xu J.-J. Ding X.-P. Xie H.-Q. Li-Ling J. (2017). Altered gene expression profile in a rat model of gentamicin-induced ototoxicity and nephrotoxicity and the potential role of upregulated Ifi44 expression. Mol. Med. Rep. 16, 4650–4658. 10.3892/mmr.2017.7150 28791351 PMC5647021

[B7] Kalinec G. M. Lomberk G. Urrutia R. A. Kalinec F. (2017). Resolution of cochlear inflammation: novel target for preventing or ameliorating drug-, noise- and age-related hearing loss. Front. Cell. Neurosci. 11:192. 10.3389/fncel.2017.00192 28736517 PMC5500902

[B8] Kapadia R. Yi J.-H. Vemuganti R. (2008). Mechanisms of anti-inflammatory and neuroprotective actions of PPAR-γ agonists. Front. Biosci. 13, 1813–1826. 10.2741/2802 17981670 PMC2734868

[B9] Karlidag T. Yalcin S. Ozturk A. Ustundag B. Gok U. Kaygusuz I.. (2002). The role of free oxygen radicals in noise induced hearing loss: effects of melatonin and methylprednisolone. Auris Nasus Larynx 29, 147–152. 10.1016/s0385-8146(01)00137-7 11893449

[B10] Kaur T. Mukherjea D. Sheehan K. Jajoo S. Rybak L. P. Ramkumar V. (2011). Short interfering RNA against STAT1 attenuates cisplatin-induced ototoxicity in the rat by suppressing inflammation. Cell Death Dis. 2:e180. 10.1038/cddis.2011.63 21776018 PMC3199718

[B11] Keithley E. M. (2018). “Cochlear inflammation associated with noise-exposure,” in Inflammatory Mechanisms in Mediating Hearing Loss, eds Ramkumar V. Rybak L. P. (Cham, Switzerland: Springer International Publishing), 91–114.

[B12] Kubota T. Matsuoka M. Xu S. Otsuki N. Takeda M. Kato A.. (2011). PIASy inhibits virus-induced and interferon-stimulated transcription through distinct mechanisms. J. Biol. Chem. 286, 8165–8175. 10.1074/jbc.M110.195255 21199872 PMC3048703

[B13] Kubota T. Matsuoka M. Chang T. H. Tailor P. Sasaki T. Tashiro M.. (2008). Virus infection triggers SUMOylation of IRF3 and IRF7, leading to the negative regulation of type I interferon gene expression. J. Biol. Chem. 283, 25660–25670. 10.1074/jbc.M804479200 18635538 PMC2533075

[B14] Li R. Pan Y. Shi D. D. Zhang Y. Zhang J. (2013). PIAS1 negatively modulates virus triggered type I IFN signaling by blocking the DNA binding activity of IRF3. Antiviral Res. 100, 546–554. 10.1016/j.antiviral.2013.09.001 24036127

[B15] Liu B. Liao J. Rao X. Kushner S. A. Chung C. D. Chang D. D.. (1998). Inhibition of stat1-mediated gene activation by PIAS1. Proc. Natl. Acad. Sci. U S A 95, 10626–10631. 10.1073/pnas.95.18.10626 9724754 PMC27945

[B16] Luu K. Greenhill C. J. Majoros A. Decker T. Jenkins B. J. Mansell A. (2014). STAT1 plays a role in TLR signal transduction and inflammatory responses. Immunol. Cell Biol. 92, 761–769. 10.1038/icb.2014.51 25027037

[B17] Maeshiba Y. Kiyota Y. Yamashita K. Yoshimura Y. Motohashi M. Tanayama S. (1997). Disposition of the new antidiabetic agent pioglitazone in rats, dogs and monkeys. Arzneimittelforschung 47, 29–35. 9037440

[B18] Martin H. (2010). Role of PPAR-gamma in inflammation. Prospects for therapeutic intervention by food components. Mutat. Res. 690, 57–63. 10.1016/j.mrfmmm.2009.09.009 20973164

[B19] Nakagawa T. (2014). Strategies for developing novel therapeutics for sensorineural hearing loss. Front. Pharmacol. 5:206. 10.3389/fphar.2014.00206 25278894 PMC4165348

[B20] Oh G.-S. Kim H.-J. Choi J.-H. Shen A. Kim C.-H. Kim S.-J.. (2011). Activation of lipopolysaccharide-TLR4 signaling accelerates the ototoxic potential of cisplatin in mice. J. Immunol. 186, 1140–1150. 10.4049/jimmunol.1002183 21148032

[B21] Orasanu G. Ziouzenkova O. Devchand P. R. Nehra V. Hamdy O. Horton E. S.. (2008). The peroxisome proliferator-activated receptor-gamma agonist pioglitazone represses inflammation in a peroxisome proliferator-activated receptor-alpha-dependent manner *in vitro* and *in vivo* in mice. J. Am. Coll. Cardiol. 52, 869–881. 10.1016/j.jacc.2008.04.055 18755353 PMC2633943

[B22] Paciello F. Fetoni A. R. Rolesi R. Wright M. B. Grassi C. Troiani D.. (2018). Pioglitazone represents an effective therapeutic target in preventing oxidative/inflammatory cochlear damage induced by noise exposure. Front. Pharmacol. 9:1103. 10.3389/fphar.2018.01103 30349478 PMC6187064

[B23] Peri F. Calabrese V. (2014). Toll-like receptor 4 (TLR4) modulation by synthetic and natural compounds: an update. J. Med. Chem. 57, 3612–3622. 10.1021/jm401006s 24188011 PMC4040398

[B24] Priuska E. M. Schacht J. (1995). Formation of free radicals by gentamicin and iron and evidence for an iron/gentamicin complex. Biochem. Pharmacol. 50, 1749–1752. 10.1016/0006-2952(95)02160-4 8615852

[B25] Roberts J. C. Friel S. L. Roman S. Perren M. Harper A. Davis J. B.. (2009). Autoradiographical imaging of PPARγ agonist effects on PBR/TSPO binding in TASTPM mice. Exp. Neurol. 216, 459–470. 10.1016/j.expneurol.2009.01.002 19320004

[B26] Rock K. L. Latz E. Ontiveros F. Kono H. (2010). The sterile inflammatory response. Annu. Rev. Immunol. 28, 321–342. 10.1146/annurev-immunol-030409-101311 20307211 PMC4315152

[B27] Sekulic-Jablanovic M. Petkovic V. Wright M. B. Kucharava K. Huerzeler N. Levano S.. (2017). Effects of peroxisome proliferator activated receptors (PPAR)-γ and -α agonists on cochlear protection from oxidative stress. PLoS One 12:e0188596. 10.1371/journal.pone.0188596 29182629 PMC5705132

[B28] Sergi B. Fetoni A. R. Paludetti G. Ferraresi A. Navarra P. Mordente A.. (2006). Protective properties of idebenone in noise-induced hearing loss in the guinea pig. Neuroreport 17, 857–861. 10.1097/01.wnr.0000221834.18470.8c 16738476

[B29] Sobkowicz H. M. Loftus J. M. Slapnick S. M. (1993). Tissue culture of the organ of corti. Acta Otolaryngol. Suppl. 502, 3–36. 10.3109/00016489309130126 8475741

[B30] Takemura K. Komeda M. Yagi M. Himeno C. Izumikawa M. Doi T.. (2004). Direct inner ear infusion of dexamethasone attenuates noise-induced trauma in guinea pig. Hear. Res. 196, 58–68. 10.1016/j.heares.2004.06.003 15464302

[B31] Vethanayagam R. R. Yang W. Dong Y. Hu B. H. (2016). Toll-like receptor 4 modulates the cochlear immune response to acoustic injury. Cell Death Dis. 7:e2245. 10.1038/cddis.2016.156 27253409 PMC5143385

[B32] Wood M. B. Zuo J. (2017). The contribution of immune infiltrates to ototoxicity and cochlear hair cell loss. Front. Cell. Neurosci. 11:106. 10.3389/fncel.2017.00106 28446866 PMC5388681

[B33] Yang C.-H. Hwang C.-F. Yang M.-Y. Lin P.-M. Chuang J.-H. (2015). Expression of toll-like receptor genes in leukocytes of patients with sudden sensorineural hearing loss. Laryngoscope 125, E382–387. 10.1002/lary.25241 25809471

[B34] Yang S. Cai Q. Vethanayagam R. R. Wang J. Yang W. Hu B. H. (2016). Immune defense is the primary function associated with the differentially expressed genes in the cochlea following acoustic trauma. Hear. Res. 333, 283–294. 10.1016/j.heares.2015.10.010 26520584 PMC4798880

[B35] Youssef J. Badr M. (2004). Role of peroxisome proliferator-activated receptors in inflammation control. J. Biomed. Biotechnol. 2004, 156–166. 10.1155/S1110724304308065 15292582 PMC551585

